# The Wnt/β-catenin signaling/Id2 cascade mediates the effects of hypoxia on the hierarchy of colorectal-cancer stem cells

**DOI:** 10.1038/srep22966

**Published:** 2016-03-11

**Authors:** Hye-Jin Dong, Gyu-Beom Jang, Hwa-Yong Lee, Se-Ra Park, Ji-Young Kim, Jeong-Seok Nam, In-Sun Hong

**Affiliations:** 1 Laboratory of stem cell research, Lee Gil Ya Cancer and Diabetes Institute, Gachon University, Incheon, 406-840, Republic of Korea; 2 Department of Molecular Medicine, School of Medicine, Gachon University, Incheon 406-840, Republic of Korea; 3 The Faculty of Liberal Arts, Jungwon University, Chungbuk, 367-805, Republic of Korea; 4 School of Life Sciences, Gwangju Institute of Science and Technology, Gwangju 500-712, Republic of Korea

## Abstract

Hypoxia, a feature common to most solid tumors, is known to regulate many aspects of tumorigenesis. Recently, it was suggested that hypoxia increased the size of the cancer stem-cell (CSC) subpopulations and promoted the acquisition of a CSC-like phenotype. However, candidate hypoxia-regulated mediators specifically relevant to the stemness-related functions of colorectal CSCs have not been examined in detail. In the present study, we showed that hypoxia specifically promoted the self-renewal potential of CSCs. Through various *in vitro* studies, we found that hypoxia-induced Wnt/β-catenin signaling increased the occurrence of CSC-like phenotypes and the level of Id2 expression in colorectal-cancer cells. Importantly, the levels of hypoxia-induced CSC-sphere formation and Id2 expression were successfully attenuated by treatment with a Wnt/β-catenin-signaling inhibitor. We further demonstrated, for the first time, that the degree of hypoxia-induced CSC-sphere formation (CD44^+^ subpopulation) *in vitro* and of tumor metastasis/dissemination *in vivo* were markedly suppressed by knocking down Id2 expression. Taken together, these data suggested that Wnt/β-catenin signaling mediated the hypoxia-induced self-renewal potential of colorectal-cancer CSCs through reactivating Id2 expression.

Local oxygen (O_2_) concentrations can directly affect the differentiation or self-renewal capacity of stem cells. Recent *in vitro* evidence indicated that hypoxia, defined as reduced oxygen tension, strongly promoted poor patient survival, therapeutic resistance and an aggressive tumor phenotype^1^. It has also been suggested that a subset of tumor cells, called cancer-stem cells (CSCs), contribute to tumor growth, metastasis, and recurrence^2^. Furthermore, CSCs have been shown to be resistant to conventional therapies, such as chemotherapy^3^ and radiation^4^. Importantly, it has also been reported that hypoxia increased the size of CSC subpopulations and promoted the acquisition of a CSC-like phenotype^5^, thereby aggravating the patient’s prognosis. This evidence of the stimulatory effects of hypoxia on tumorigenesis prompted us to investigate the underlying mechanisms by which hypoxia regulates the tumorigenic properties of CSCs in colorectal cancers.

In promoting colorectal-cancer development, several target genes that directly respond to hypoxia have been identified, including those involved in Wnt/β-catenin signaling^6^. This well-characterized signaling pathway is one of the most important potential regulators of tumorigenesis in many different types of solid tumors, such as ovarian^7^, colorectal^8^, and breast cancers^9^. Aberrant Wnt/β-catenin signaling is a key regulator of colorectal-cancer development and is known to contribute to early events in the progression of colorectal cancer^10^. However, less is known about the potential mediators responsible for the tumorigenic effects of Wnt/β-catenin signaling under a hypoxic condition.

As a first step in uncovering specific mediators that control the maintenance of CSC self-renewal potential under a hypoxic condition, we initially focused on genes that were previously implicated in the self-renewal properties of embryonic or somatic stem cells under a hypoxic condition, then further narrowed down the list of potential candidate genes to those that were shown to be tightly regulated by Wnt/β-catenin signaling in various types of human cancers, and finally further reduced the list to those mainly related specifically to the development of colorectal cancer. One of the candidate genes that satisfied all these requirements encodes inhibitor of DNA-binding (Id) proteins, a family of helix-loop-helix (HLH) transcriptional regulatory proteins^11^. The Id proteins are essential for embryogenesis/organogenesis, and they have been functionally implicated in basic cellular processes, such as cell proliferation, apoptosis, and differentiation^12^,^13^. The Ids are not generally found in terminally differentiated cells. However, the expression of Id proteins is reactivated in many different types of cancer. For example, Id expression has been documented in breast^14^, bladder^14^, colon^15^, pancreatic^16^ and prostate cancers^17^, as well as in T-cell lymphomas^18^. Consistent with these findings, the increased expression of Id proteins induces cell proliferation and metastasis, and thus these proteins can be used as useful prognostic or predictive markers for many different types of human cancers^13^,^19^,^20^. More recent studies found a direct relationship between the Id proteins and hypoxia during tumor development^21^,^22^. Interestingly, recent studies showed that the expression of only one of the Id protein family members, Id2, was up-regulated in terminally differentiated cell types following hypoxia-induced injury^23^,^24^,^25^. Thus, it is quite possible that a functional connection between colorectal CSCs and deregulated Id2 expression exists to regulate hypoxia-mediated tumorigenesis. Consistent with this hypothesis, the reactivated expression of Id2 has been demonstrated in highly malignant pancreatic^16^ and colorectal cancers^26^,^27^.

Here, we showed that hypoxia specifically up-regulated CSC sphere formation and the size of a subset of CD44^+^ CSC subpopulations. Through various *in vitro* studies, we found that hypoxia-induced Wnt/β-catenin signaling increased the occurrence of CSC-like phenotypes and the level of Id2 expression. To better understand the role of Id2 in colorectal cancers, we used shRNA to establish a stable Id2 knock-down cell line, and found that these cells had an increased rate of apoptosis and a reduced growth potential. Evaluation of their metastatic potential demonstrated that Id2 depletion was accompanied by a decreased level of migration across a transwell membrane and decreased levels of the critical migration regulators MMP2 and MMP9. We further demonstrated, for the first time, that hypoxia-induced CSC sphere formation (CD44^+^ subpopulation), and the expression of stem-cell markers *in vitro* and tumor metastasis/dissemination *in vivo* were markedly suppressed by knocking down Id2 expression. Importantly, the levels of hypoxia-induced CSC sphere formation and Id2 expression were successfully attenuated by treatment with a Wnt/β-catenin-signaling inhibitor. Taken together, these data suggested that Wnt/β-catenin signaling mediated the hypoxia-induced reactivation of Id2 expression and consequently, the enhanced level of Id2 promoted the self-renewal potential of CSCs and tumor metastasis/dissemination as a downstream effector of hypoxia-induced Wnt/β-catenin signaling during colorectal-cancer development.

## Results

### Wnt/β-catenin signaling was activated by hypoxia of colorectal cancer cells

**Figure 1:**
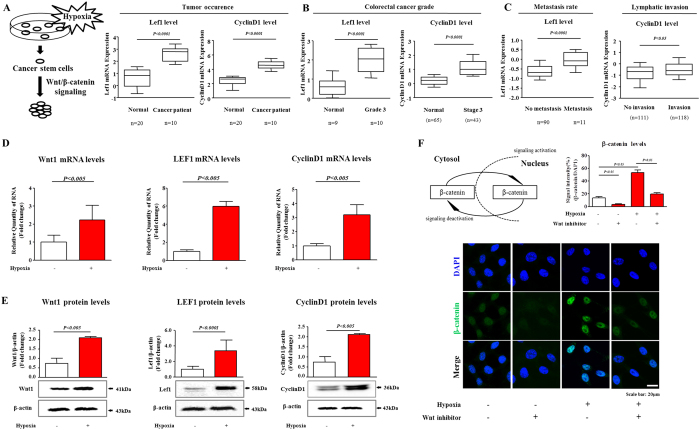
The effects of hypoxia on Wnt/β-catenin signaling in colorectal-cancer cells. (**A–C**) A significant correlation between tumor development/metastasis and the expression of Wnt/β-catenin-signaling components was observed in the human colorectal-cancer datasets available through the Oncomine dataset repository (www.oncomine.org). Real-time PCR (**D**) and western blotting (**E**) demonstrated the hypoxia-induced changes in the expression of Wnt/β-catenin-signaling components (Wnt1, Lef1, and cyclin D1). (**F**) Colorectal-cancer cells were stained using an antibody specific for β-catenin. The stimulatory effects of hypoxia on these activities of β-catenin were successfully attenuated after Wnt/β-catenin-signaling inhibitor treatment. DAPI staining was used to label the nuclei. β-actin was used as the internal control. The results are the mean values ± SD from three independent experiments.

Recently, accumulating evidence has demonstrated the critical role of Wnt/β-catenin signaling in various CSCs^28^. Thus, to investigate the association between the development and prognosis of colorectal cancer and Wnt/β-catenin signaling, we evaluated the available colorectal-cancer datasets in the Oncomine dataset repository (www.oncomine.org). After specifically filtering for datasets obtained from colorectal cancer with an invasive phenotype, we observed significant correlations between tumor development/metastasis and the expression of components of Wnt/β-catenin signaling (Fig. 1A–C). Previous studies demonstrated that under hypoxic conditions, the aberrant activation of Wnt/β-catenin signaling was associated with the development of a variety of tumor types^29^,^30^. To investigate the effect of hypoxia on Wnt/β-catenin signaling in colorectal-cancer cells, we thus examined the quantitative changes in the expression of positive (Wnt1, Lef1, cyclin D1) regulators of Wnt/β-catenin signaling with or without hypoxia. To induce hypoxia, cells were cultured in hypoxia chambers (1% O_2_). We conducted the additional sets of experiments to determine which HIF factor is involved in hypoxia-mediated effects. Interestingly, hypoxia consistently increased HIF1α expression, but not HIF2α, in all three additional independent experiments (Supplementary Fig. 1A,B). The mRNA (Fig. 1D) and protein (Fig. 1E) levels of the positive regulators of Wnt/β-catenin signaling were markedly increased in the colorectal-cancer cells under hypoxic conditions. The increased stability of β-catenin following Wnt/β-catenin signaling leads to its translocation into the nucleus, where it induces the transcriptional activation of target genes^31^. Therefore, to further assess the significance of Wnt/β-catenin signaling activity during hypoxia, we examined the localization of β-catenin upon hypoxia with or without Wnt/β-catenin-signaling inhibitor. Consistent with the results described above, the hypoxic exposure increased the nuclear localization and expression of β-catenin in the colorectal-cancer cells. The stimulatory effects of hypoxia on these activities of β-catenin were successfully attenuated after Wnt/β-catenin-signaling inhibitor treatment in the colorectal-cancer cells (Fig. 1F). To further evaluate the hypoxia-mediated enhancement of β-catenin expressions, we performed real-time PCR and western blotting to quantitate the levels of β-catenin with or without hypoxic exposure. As expected, hypoxia significantly increased both RNA and protein levels for β-catenin (Supplementary Fig. 2A,B).

### The Wnt/β-catenin-signaling inhibitor suppressed the hypoxia-induced development of immature phenotypic characteristics

It has been suggested that the three-dimensional (3D) spheres formed *in vitro* were enriched for the cancer stem/progenitor cells of different types of cancers, including breast^32^, colon^33^, and pancreatic cancer^34^. Recent studies suggested that the stem-cell markers c-Myc^35^ and Sox2^36^ play important roles in maintaining the pluripotency of colorectal CSCs. Here, we established a sphere-formation culture system as an *in vitro* CSC-culture model using our published protocols^37^.

**Figure 2:**
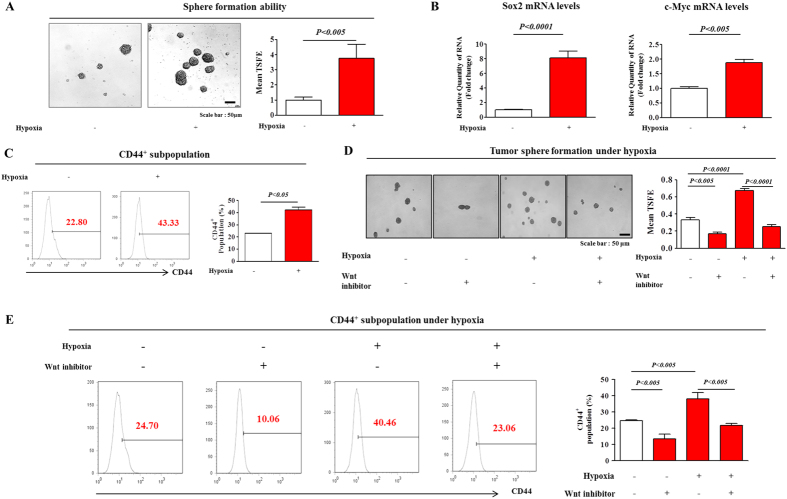
Stimulatory effect of hypoxia on the growth and the stemness-related characteristics of colorectal CSCs. (**A**) Hypoxia stimulated CSC-sphere formation by the mouse colorectal-cancer cells after 4 days under sphere-formation culturing conditions. The number of spheres that were larger than 150 μm in diameter was enumerated, and a representative image of the CSC-containing spheres is shown. The data represents the average values from three independent experiments. (**B**) Real-time PCR data demonstrated the hypoxia-induced enhanced expression of the stem-cell markers c-Myc and Sox2. (**C**) The results of FACS analysis showed the increased percentage of CD44-positive cells among the total population of mouse colorectal-cancer cells grown under the hypoxic condition. (**D**) The stimulatory effects of hypoxia on colorectal CSC-sphere formation were successfully attenuated by treatment with a Wnt/β-catenin-signaling inhibitor. (**E**) Treatment with a Wnt/β-catenin-signaling inhibitor led to the decrease in the percentage of CD44-positive cells in the colorectal-cancer cells grown under hypoxic conditions. The results are the mean values ± SD from three independent experiments.

Consistent with the results of previous studies, the levels of expression of stem-cell markers (Klf4, Oct4, and Sox2) were higher in the sphere-forming cells than in the cells in monolayers (Supplementary Fig. 3A–D). To determine whether sphere-forming subpopulations and cells with stem cell-like properties were enriched under hypoxic conditions, we evaluated the effect of hypoxia on sphere formation and the expression profiles of the stem cell markers c-Myc and Sox2. The number and size of the spherical colonies were significantly increased by hypoxia of the colorectal-cancer cells (Fig. 2A). Consistent with the sphere-formation results, the levels of expression of the stem-cell markers were increased in the hypoxic cells compared with those of the normoxic cells (Fig. 2B). It has also been reported that CD44^+^ cell populations (thought to be stem cell-like) were enriched in colorectal tumorigenic stem/progenitor cells^38^. We therefore performed FACS analysis to quantify the percentage of the CD44^+^ cell population in both hypoxic and normoxic colorectal-cancer cells. As expected, the relative percentage of cells expressing CD44 was markedly increased in the hypoxic cells compared with that of the normoxic cells (Fig. 2C). Moreover, we further confirmed the effects of hypoxia on the expression of cancer stemness related factors and several key components of Wnt/β-catenin signaling with human colonic carcinoma epithelial cell line HCT116. As expected, hypoxic exposure significantly increased cancer stemness related factors (c-Myc, Klf4, Nanog, and Oct4) (Supplementary Fig. 4A) and Wnt/β-catenin signaling components (Wnt1, TCF4, and Cyclin D1) (Supplementary Fig. 4B,C). Therefore, it was reasonable to hypothesize that hypoxia stimulated the growth of CSCs through activating the Wnt/β-catenin signaling pathway in colorectal-cancer cells. Firstly, we tested the efficacy and specificity of ICG-001 to inhibit Wnt/β-catenin signaling in CT26 cells transiently transfected with a luciferase reporter plasmid in the presence or absence of lithium chloride (LiCl), an activator of the Wnt/β-catenin signaling. In response to ICG-001 treatment, LiCl-induced transcriptional activities were significantly attenuated in a dose-dependent manner (Supplementary Fig. 5A). An approximate IC_50_ value of the Wnt/β-catenin-signaling inhibitor was determined from a dose-response curve. The IC_50_ value of the colorectal-cancer cells was 2.676 μM (Supplementary Fig. 5B). Treatment with the Wnt/β-catenin-signaling inhibitor inhibited the hypoxia-induced CSC-sphere formation of the colorectal-cancer cells (Fig. 2D). We hypothesized that inhibiting Wnt/β-catenin signaling might disrupt CSC sphere formation by targeting the CD44^+^ subpopulations. To test this hypothesis, we used FACS analysis to investigate the effect of the Wnt/β-catenin-signaling inhibitor on the CD44^+^ subpopulations. Indeed, treating the colorectal-cancer cells with the Wnt/β-catenin-signaling inhibitor for 48 h decreased the size of the CD44^+^ subpopulation (Fig. 2E). Furthermore, to determine whether Hif1α-specific inhibition affects the stemness of CSCs, we also performed FACS analysis to quantitate the percentage of the CD44^+^ population with or without Hif1α-specific inhibitor treatment. Consistent with upper results, the percentage of cells with this CD44^+^ subpopulation was markedly reduced in treated-cells than non-treated cells (Supplementary Fig. 6A). Therefore, it is quite reasonable to assume that hypoxia-mediated Wnt/Id2 cascade regulates the stemness of CSCs through Hif1α activity.

### The stimulatory effects of hypoxia on CSCs were achieved through the up-regulation of Id2 expression

**Figure 3:**
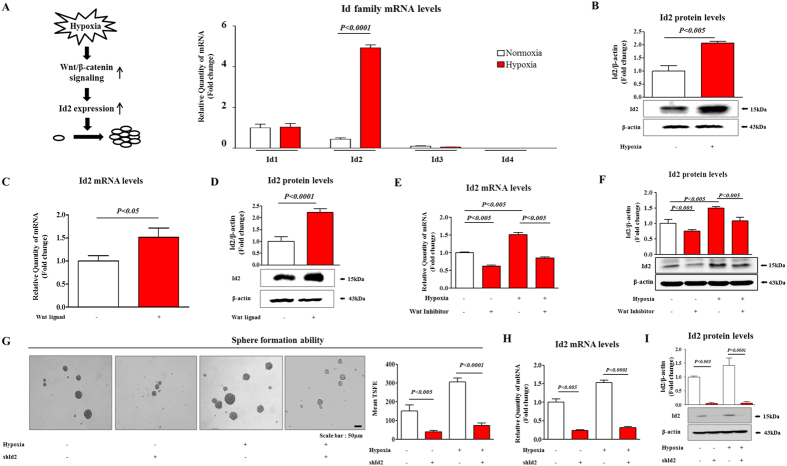
Knocking down Id2 expression suppressed the hypoxia-induced increased growth and exhibition of stemness-related characteristics of colorectal CSCs. (**A**) Mouse colorectal-cancer cells cultured under the hypoxic condition were evaluated for the expression level of Id family members (Id1-Id4). (**B**) The stimulatory effect of hypoxia on the expression of Id2 was verified by western blotting analysis. (**C**) The results of real-time PCR and (**D**) western blotting assays demonstrated the change of Id2 expression that significantly increased by Wnt-ligand treatment. The levels of expression of Id2 in mouse colorectal-cancer cells treated with the Wnt/β-catenin-signaling inhibitor (2 μM) with or without hypoxia were evaluated using (**E**) real-time PCR and (**F**) western blotting. (**G**) Knocking down Id2 expression successfully attenuated the hypoxia-induced CSC-sphere formation. The number of spheres that were larger than 150 μm in diameter was counted, and a representative image of a tumorsphere is shown. The Id2 knockdown was verified based on the (**H**) mRNA and (**I**) protein levels observed under our colorectal CSC-culturing conditions. β-actin was used as the internal control. Abbreviations: TSFE, tumor sphere-forming efficiency. The results are the mean values ± SD from three independent experiments.

The Ids are essential for embryogenesis, and they have been functionally implicated in many human cancers, including breast^14^, bladder^14^, colon^15^, and pancreatic cancer^16^. Recent studies established a direct connection between the Ids and hypoxia during tumor development^21^,^22^. Therefore, it was quite reasonable to hypothesize that the Ids mediate hypoxia-induced tumor growth and promote CSC renewal as downstream effectors of hypoxia during colorectal-cancer development. To investigate the potential role of hypoxia on the expression of Id family members in colorectal cancers, we analyzed the levels of expression of Id family members under the hypoxic condition. The expression level of Id2 but not that of Id1, 3 or 4 was increased in hypoxic cells compared with that of normoxic cells in (Fig. 3A). Consistent with the qPCR data, the expression level of Id2 was significantly increased in colorectal-cancer cells under the hypoxic condition (Fig. 3B), suggesting that hypoxia was highly associated with enhanced Id2 expression in colorectal cancers. To further investigate the potential role of hypoxia in Id2 expression and the regulatory role of Id2 in hypoxia-induced Wnt/β-catenin signaling in colorectal cancer, we analyzed the level of Id2 expression in the presence or absence of Wnt ligands under the hypoxic condition. Importantly, the mRNA and protein levels of Id2 were significantly increased by Wnt-ligand treatment (Fig. 3C,D). As expected, pre-treating the cells with the Wnt/β-catenin-signaling inhibitor strongly decreased the hypoxia-induced up-regulation of Id2 expression (Fig. 3E,F). Additionally, to determine whether Hif1α-specific inhibition affects Wnt/β-catenin signaling and subsequent Id2 expression, we also analyzed the mRNA level of Hif1α, Wnt/β-catenin signaling component TCF4, and Id2 with or without Hif1α inhibitor (Topotecan). As expected, the mRNA levels of these factors were significantly decreased in Hif1α specific inhibitor-treated cells compared with non-treated cells (Supplementary Fig. 7A). To further confirm the potential role of hypoxia in Id2 expression and hypoxia-induced CSC-sphere formation in human colonic carcinoma epithelial cell line HCT116, we analyzed the level of Id2 expression in the presence or absence of Wnt/β-catenin-signaling inhibitor (ICG-001) with or without hypoxic exposure. Consistent with the results from CT26 cells, pre-treating HCT116 cells with ICG-001 strongly decreased the hypoxia-induced sphere formation (Supplementary Fig. 7B) and Id2 expression at both the mRNA and protein levels (Supplementary Fig. 7C,D). Taken together, these data suggested that hypoxia-induced Wnt/β-catenin signaling was strongly associated with enhanced Id2 expression in colorectal cancer. Therefore, to investigate whether Id2 depletion was sufficient to inhibit the hypoxia-induced CSC growth, we knocked down Id2 expression in colorectal-cancer cells using a specific shRNA. Cell lines with stable knocked-down Id2 expression were established using a lentiviral system (Supplementary Fig. 8A–C). As a functional assay, we evaluated the effect of the Id2 knockdown on CSC sphere formation. The stimulatory effects of hypoxia on CSC-sphere formation were successfully attenuated by the Id2 knockdown in colorectal-cancer cells (Fig. 3G). The successful knockdown of Id2 expression was verified based on the RNA (Fig. 3H) and protein levels (Fig. 3I) under our colorectal CSC-culture conditions. To test whether the level of Id2 expression was increased under the three-dimensional culture conditions, we examined the levels of Id2 mRNA and Id2 protein. The expression level of Id2 but not that of Id1, 3 or 4 was higher in the sphere-forming cells than in the cells in monolayers (Supplementary Fig. 9A,B). These results suggested that the stimulatory effects of hypoxia on the CSCs were achieved through the up-regulation of Id2 expression.

### Id2 depletion reduced the rate of tumor growth in a murine xenograft model

**Figure 4:**
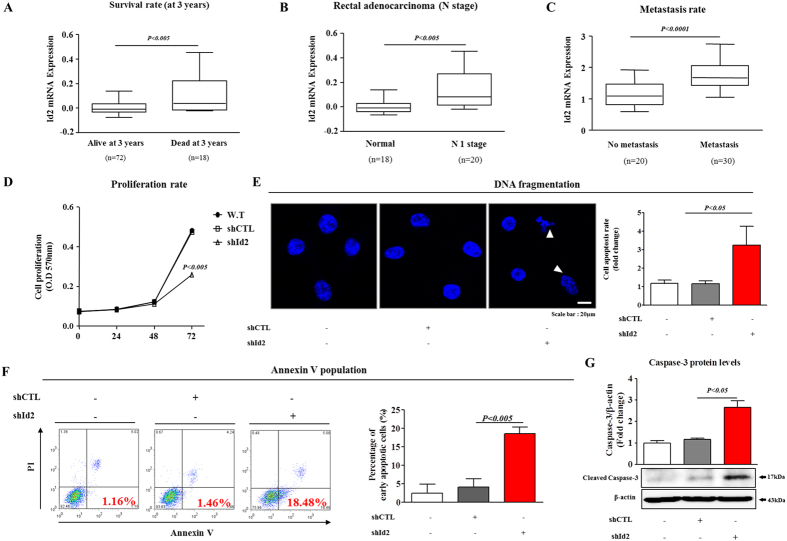
The effects of the Id2 knockdown on colorectal cancer-cell growth and apoptosis. (**A–C**) A significant correlation between tumor development/metastasis and Id2 expression were observed in human colorectal-cancer datasets available through the Oncomine dataset repository (www.oncomine.org). (**D**) Transfection of colorectal-cancer cells with Id2 shRNA led to a time-dependent decrease in the number of cells compared with transfection with the control shRNA. (**E**) Id2 knockdown-mediated apoptotic DNA fragmentation and condensation were visualized using DAPI staining. (**F**) The Id2 knockdown-mediated cytotoxicity was evaluated by flow cytometry using PE-labeled anti-annexin-V. (**G**) The level of activated (cleaved) caspase 3 in the cells undergoing Id2 knockdown-induced apoptotic death was evaluated by western blotting using an antibody directed against activated caspase 3. DAPI staining was used to label the nuclei. β-actin was used as the internal control. The results are the mean values ± SD from three independent experiments.

To investigate the association between tumor development/metastasis and Id2 expression, we evaluated the available colorectal-cancer datasets in the Oncomine dataset repository (www.oncomine.org). After specifically filtering for the datasets of colorectal cancers showing tumor recurrence or metastasis, we observed significant correlations between upregulated Id2 expression and a higher incidence of colorectal-cancer recurrence or metastasis (Fig. 4A–C). To assess the effect of the Id2 knockdown on the growth of colorectal-cancer cells, cell viability was determined using an MTT assay. As shown in Fig. 4D, a time-dependent decrease in the number of Id2-knockdown cells was observed compared with the control shRNA-infected cells. Apoptotic cell death was qualitatively estimated using DAPI staining to reveal nuclear condensation and fragmentation. The Id2 knockdown led to significant DNA fragmentation compared with that of the shRNA control cells (Fig. 4E). A flow cytometric assay using a PE-labeled annexin-V antibody was used to further evaluate the effect of the Id2 knockdown on apoptosis. The apoptotic rate of colorectal cells transfected with Id2 shRNA reached 18.48%, whereas the apoptotic rate of control shRNA-transfected cells was 1.46% (Fig. 4F). Western blotting revealed that the Id2 knockdown induced the activation of caspase-3 (an indicator of apoptosis) (Fig. 4G).

**Figure 5:**
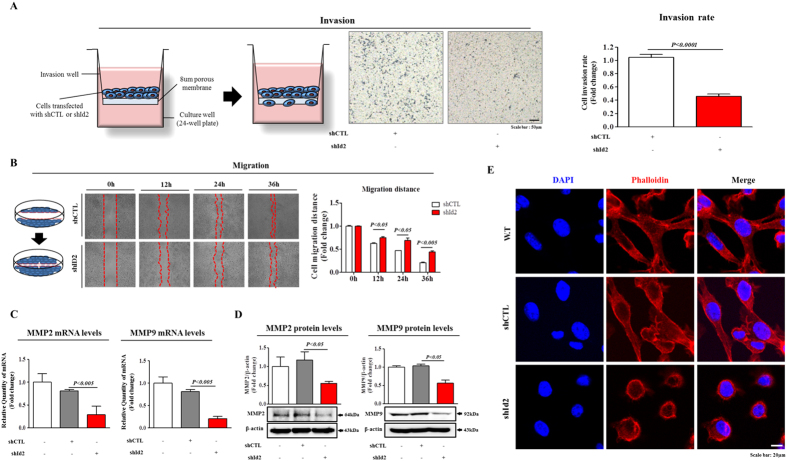
The effects of the Id2 knockdown on the invasion and migration of colorectal-cancer cells. (**A**) The cell-invasion ability of the colorectal-cancer cells was evaluated using a transwell assay. Transfection with Id2 shRNA significantly decreased the degree of their invasion across the transwell membrane compared with transfection with the control shRNA. (**B**) The effects of the Id2 knockdown on the migration of colorectal-cancer cells were evaluated using a scratch assay. The migration of cells transfected with the shRNA targeting Id2 was slower than that of the cells transfected with the control nontargeting shRNA. (**C**,**D**) The relative level of expression of the migration regulators MMP2 and MMP9 was assessed using real-time PCR and western blot. (**E**) The Id2 knockdown-induced disorganization of actin filaments and the morphological transition of the cells were visualized through phalloidin staining of actin filaments. DAPI staining was used to label the nuclei. β-actin was used as the internal control. The results are the mean values ± SD from three independent experiments.

We also investigated the roles of Id2 in the invasion and migration of colorectal-cancer cells using a transwell migration assay and a scratch assay. The results showed that following the Id2 knockdown, the ability of these cells to migrate across the transwell membrane was significantly decreased (Fig. 5A). Additionally, the migration of cells transfected with the shRNA targeting Id2 was slower than that of the cells transfected with control non-targeting shRNA (Fig. 5B). To confirm the stimulatory effect of Id2 on the migration of colorectal-cancer cells, western blotting was used to evaluate the expression levels of MMP-2 and MMP-9, which play an important role in regulating migration. The Id2-knockdown cells had a significantly decreased level of MMP-2/9 expression compared with that of the control shRNA-transfected cells (Fig. 5C,D). These results suggested that Id2 was necessary for migration and therefore might play roles in colorectal-cancer metastasis. Previous studies indicated that the actin cytoskeleton was required for tumor cell migration via pushing or pulling on the substrate near the plasma membrane^39^. Therefore, we examined the distribution of the actin cytoskeleton at the subcellular level in colorectal-cancer cells following the Id2 knockdown. Phalloidin staining of actin filaments revealed a strong correlation between the Id2 knockdown and a highly disorganized actin cytoskeleton (Fig. 5E), suggesting that the reduced migration of the Id2-knockdown cells may be related to the disorganization of the actin cytoskeleton. Furthermore, we also investigated whether the effects of Id2 knockdown on the metastasis, cell proliferation, and migration were caused, or at least partially caused by Id2 shRNA-induced apoptosis. We therefore investigated whether Id2-induced cell death is dependent on caspase-mediated activation, CT26 cells were pre-incubated with the broad-spectrum caspase inhibitor Z-VAD-FMK with or without Id2 shRNA transfection. Pretreatment of cells with Z-VAD-FMK successfully attenuated Id2-induced caspase-3,8 and PARP cleavage, suggesting that Id2 knockdown induces a caspase-dependent apoptosis at least partly in CT26 cells (Supplementary Fig. 10A). In a next step, we applied Z-VAD-FMK to further investigate whether the various Id2 knockdown-mediated effects are caused by Id2 shRNA-induced apoptotic cell death. Interestingly, we have found that Z-VAD-FMK pre-treatment attenuated Id2 knockdown-mediated effects on the proliferation (Supplementary Fig. 10B) and migration (Supplementary Fig. 10C), indicating that Id2 shRNA-induced apoptosis may affect, or at least partially affect the various Id2 knockdown-mediated effects.

**Figure 6:**
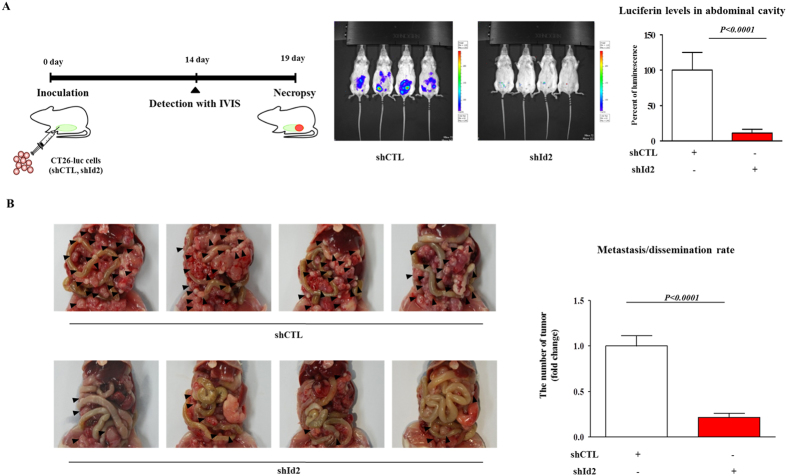
The effects of knocking down Id2 expression in colorectal-cancer cells on their metastasis/dissemination (**A**,**B**) Mouse colorectal-cancer cells expressing firefly luciferase (CT26-Luc) were subcutaneously injected into BALB/c mice. After their injection, the abdominal metastasis/dissemination was monitored using an IVIS; the photon intensity, a measurement of the dissemination of viable tumor cells, was also determined by the system. The results are the mean values ± SD from three independent experiments.

Following our *in vitro* experiments, we investigated the *in vivo* efficacy of the Id2 knockdown on cancer-cell dissemination using a mouse model. Colorectal-cancer cells were transfected with the firefly-luciferase gene (Supplementary Fig. 11A,B). The photon counts, as determined using an IVIS imaging system, were highly correlated to the number of disseminated cells in each group. Id2-knockdown colorectal-cancer cells were subcutaneously injected into BALB/c mice, and tumor-cell metastasis/dissemination was monitored. After injecting these cancer cells (CT26-Luc cells), their abdominal metastasis/dissemination was monitored using an IVIS; the photon intensity, a measurement of viable tumor-cell dissemination, was also determined using this system. Importantly, there was a consistent and significant reduction in the extent of tumor-cell metastasis/dissemination of the Id2-knockdown cells in the mice compared with that of the control cells, indicating that the Id2 knockdown significantly impaired the metastatic potential of the CSCs (Fig. 6A,B).

## Discussion

Deciphering the fundamental biological mechanisms underlying hypoxia-mediated tumor progression has been a major area of cancer research because the size of most solid cancers is restricted to 2–3 mm^3^ in the absence of angiogenesis and vascularization^40^. Hypoxia, a feature of most solid tumors, is known to regulate multiple aspects of tumorigenesis and is typically associated with changes such as drug resistance, metastasis/invasion, and ultimately poor clinical outcomes^41^. However, the hypoxia-regulated candidate mediators specifically relevant to the stemness-related functions of colorectal CSCs have not been examined in detail.

The present study identified Wnt/β-catenin signaling as a hypoxia-responsive regulator in colorectal-cancer cells. Consistent with this result, this signaling mode has been found to be one of the major contributors to tumorigenesis and the more aggressive phenotypes of many types of cancer. Wnt/β-catenin signaling is aberrantly upregulated in the majority of colorectal cancers^42^. Wnt/β-catenin signaling has also been demonstrated to play a major role in the regulation of stem-cell fate, and aberrant Wnt/β-catenin signaling contributed to the maintenance and *in vivo* tumorigenicity of CSCs^43^. Indeed, we provided strong evidence that *in vitro* exposure to hypoxia, defined as 1% oxygen, increased Wnt/β-catenin-signaling activity in colorectal-cancer cells (Fig. 1A–F) and therefore further enhanced the self-renewal capacity of CSCs and the expression of colorectal-CSC markers (Fig. 2A–C). These changes were accompanied by increased levels of expression of c-Myc^35^ and Sox2^36^, which are known stemness-associated markers of colorectal CSCs, suggesting a key role for hypoxia-induced Wnt/β-catenin signaling in maintaining the cancer stem-cell state in colorectal cancers. Importantly, the stimulatory effects of hypoxia on the CSC self-renewal capacity (Fig. 2D) and CSC marker expression (Fig. 2E) were significantly attenuated by treatment with a Wnt/β-catenin-signaling inhibitor, suggesting that Wnt/β-catenin signaling was necessary for the hypoxia-induced enhanced colorectal-CSC self-renewal capacity.

Tumor development is tightly regulated through the coordinated modulation of gene expression by proteins called basic helix-loop-helix (bHLH) that belong to a family of transcriptional regulators, which regulate the tumor growth, vascularization, and metastasis of various types of cancer^44^. Of particular interest is the finding that high Id expression levels were found in proliferative, undifferentiated tumor cells. To date, four distinct members of the mammalian Id protein family have been described, including Id1, Id2, Id3, and Id4^45^,^46^. The Ids are essential for embryogenesis, and they have been functionally implicated in many human cancers, including breast^14^, bladder^14^, colon^15^, and pancreatic cancer^16^. Recent studies have established a direct connection between Ids and hypoxia in tumor development^21^,^22^. The expression of only one of the Id protein family members, Id2, was induced by hypoxia (Fig. 3A,B) and by Wnt-ligand treatment (Fig. 3C,D). In this study, we also found that hypoxia-induced expression of Id2 was successfully attenuated by treatment with a Wnt/β-catenin-signaling inhibitor (Fig. 3E,F), indicating that Wnt/β-catenin signaling regulated Id2 expression during hypoxia-mediated tumorigenesis. Thus, it is possible that functional cross-talk between Wnt/β-catenin signaling and ID2 expression regulates the hypoxia-mediated tumorigenic potential of colorectal CSCs. Consistent with this hypothesis, the stimulatory effects of hypoxia on CSC-sphere formation were successfully attenuated by knocking down Id2 expression in colorectal-cancer cells (Fig. 3G–I). These results suggested that the stimulatory effects of hypoxia on CSCs were achieved through the up-regulation of Id2 expression. Generally, the expression of Id genes is detected in various proliferative undifferentiated cells *in vivo* during normal development, and the expression levels of these proteins are significantly decreased in terminally differentiated mammalian cells *in vitro or in vivo*^47^. Interestingly, recent studies have shown that Id2 expression was up-regulated in terminally differentiated cell types following hypoxia-induced injury^23^,^24^,^25^. Similarly, in the present study, the significant up-regulation of Id2 expression in colorectal-cancer cells after hypoxic exposure was demonstrated *in vitro* (Fig. 3A,B). Furthermore, we investigated whether the up-regulation of Id2 expression was associated with the rate of apoptosis of colorectal-cancer cells. Western blotting revealed that the Id2 knockdown induced the activation of caspase-3 (an indicator of apoptosis) (Fig. 4G). In addition, it was obvious that the apoptotic rate of the colorectal cells transfected with the Id2 shRNA reached 18.48%, whereas this rate was 1.46% in the control shRNA-transfected cells (Fig. 4F). Altogether, these results indicated that the Id2 expression level might be inversely correlated with colorectal cancer-cell survival. However, the precise mechanism by which Id2 regulates the survival or apoptosis of colorectal-cancer cells is not known, though many possibilities exist. It has been shown that Id2 preferentially dimerized with members of the E2A family of transcription factors and consequently prevented these proteins from binding to DNA and activating their target genes^48^,^49^. The E2A transcription factors are known to suppress cell growth by increasing the level of expression of cell-cycle inhibitors, such as the cyclin-dependent kinase inhibitor p21^(Cip1)^^50^, and potentially, senescence-associated cell cycle inhibitor p16INK4a^51^. Suppressed Id2 expression could lead to the release of E2A, enhancing the E2A-mediated suppression of cell growth. It was also previously reported that Id2 may directly interact with the cell-cycle regulator Rb^52^,^53^. The direct interaction of Rb and Id2 lead to the up-regulated expression of genes that drive cells from the S phase through the G1 phase.

In addition to its involvement in cell apoptosis and survival, Id2 has also been implicated as an important regulator of cell migration^54^, although the precise molecular mechanisms underlying this process are not completely understood. Strikingly, Coma *et al.* showed that the aberrantly elevated amount of Id2 repressed the transcriptional repression of semaphoring 3F and, as a consequence, enhanced the ability of tumor cells to migrate and invade^55^. Consistent with these results, we observed that the Id2-knockdown cells had a significantly decreased ability to migrate across the transwell membrane (Fig. 5A,B) and a significantly decreased level of MMP-2/9 expression (Fig. 5C,D) compared with those of the control shRNA-transfected cells. After completing our *in vitro* experiments, we investigated the *in vivo* effect of Id2 knockdown on tumor metastasis/dissemination using a mouse model. Importantly, there was a consistent and significant reduction of metastasis/dissemination in the mice injected with Id2-knockdown cells compared with those injected with the control cells (Fig. 6A,B). However, the inhibitory effects of the Id2 knockdown on apoptosis and migration could not completely explain the significant decrease in the *in vivo* dissemination rate observed in the Id2 knocked-downed group. The ID proteins play a pivotal role in maintaining cells in an “immature” state, a finding that is consistent with the decreased self-renewal potential of Id2-knockdown colorectal CSCs (Fig. 3G). Previous studies linked Id protein expression and certain properties of CSCs, such as self-renewal and tumor initiation. Emerging evidence indicates that the increased expression of Id1 and Id3 is positively correlated with the self-renewal and tumor-initiation abilities of colorectal cancer-stem cells^56^. Moreover, CSCs have an increased resistance to radiation and standard chemotherapeutic drugs and, in accordance with this phenomenon, the depletion of Id1 and Id3 sensitized CSCs to chemotherapeutic drugs^56^. However, the specific role of Id2 in the self-renewal and tumor initiation of colorectal CSCs is largely unexplored. Our data supported the finding of these prior studies related to the Id proteins and documented for the first time that Id2 regulated the self-renewal potential of CSCs and, furthermore, mediated the metastatic/dissemination potential of colorectal-cancer cells.

**Figure 7:**
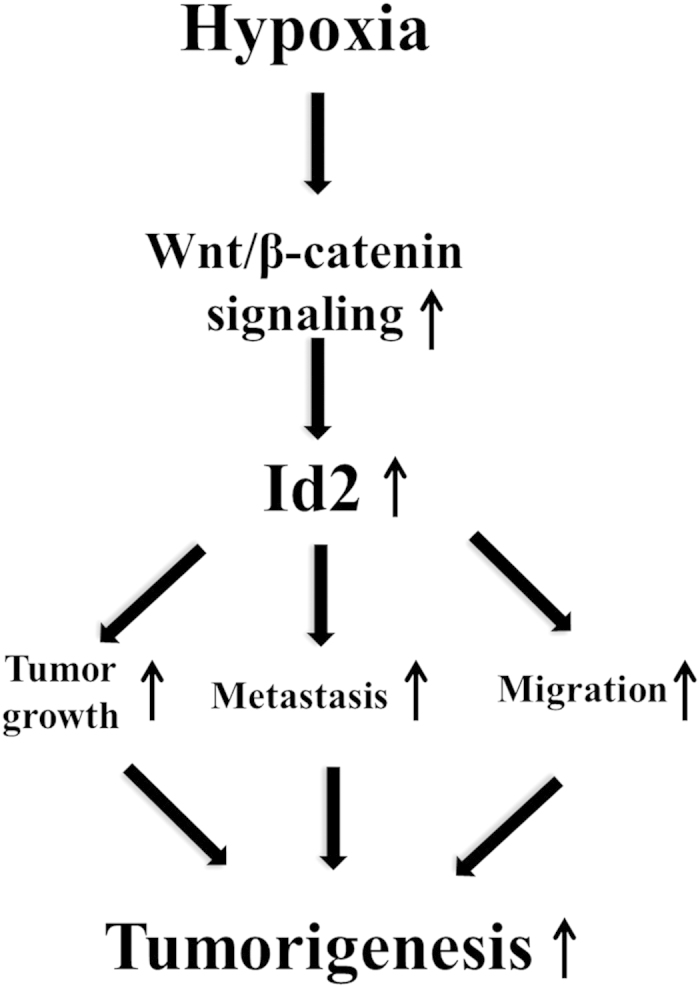
Schematic summary of the role of the Wnt/β-catenin signaling/Id2 cascade in the development of colorectal cancer under hypoxia. The hypoxia-induced enhancement of the Wnt/β-catenin signaling/Id2 cascade up-regulates the self-renewal and migration abilities of colorectal CSCs, thereby promoting the growth and dissemination of colorectal cancer cells.

In conclusion, we showed that hypoxia specifically up-regulated CSC-sphere formation and a size of a subset of the CD44^**+**^ CSC subpopulations Through various *in vitro* studies, we found that hypoxia-induced Wnt/β-catenin signaling increased the occurrence of CSC-like phenotypes and the level of Id2 expression in colorectal- cancer cells. We further demonstrated, for the first time, that the hypoxia-induced CSC-sphere formation (by a CD44^**+**^ subpopulation) *in vitro* and tumor metastasis/dissemination *in vivo* were markedly suppressed by knocking down Id2 expression. Importantly, hypoxia-induced CSC-sphere formation and Id2 expression were successfully attenuated by treatment with a Wnt/β-catenin-signaling inhibitor. Taken together, these data suggested that Wnt/β-catenin signaling mediated the hypoxia-induced reactivation of Id2 expression and, consequently, the increased level of Id2 promoted the self-renewal potential of CSCs and tumor metastasis/dissemination as a downstream effector of hypoxia-induced Wnt/β-catenin signaling during colorectal cancer development (Fig. 7).

## Methods

### Cell culture and reagents

The colon carcinoma cell lines CT26 and HCT116 were cultured in DMEM and RPMI1640 (Invitrogen, Grand Island, NY) supplemented with 10% fetal bovine serum (FBS; Gibco, Grand Island NY), 100 U/ml penicillin and 100 U/ml streptomycin (Lonza, Basel, Switzerland) at 37 °C and 5% CO_2_. Wnt signaling inhibitor ICG-001 is designed by JW Pharmaceutical Corporation (Seoul, Korea).

### Short hairpin RNA

Small hairpin RNA (shRNA) targeting mouse Id2 and non-targeting RNA were purchased from Sigma (St. Louis, MO, USA). For the efficient Id2 shRNA transfection, transfection was performed using Lipofectamine 2000 (Invitrogen) according to the manufacturer’s instructions. We chose the Id2 shRNA that is most effective in mRNA levels from five shRNA designed from the target sequence and determined by qRT-PCR.

### Tumorsphere formation

Single cells were resuspended in serum-free DMEM (Invitrogen) containing B27 (Invitrogen), 20 ng/ml EGF, 20 ng/ml bFGF (PeproTech) and 4 μg/ml heparin (Sigma-Aldrich). Primary tumorspheres were derived by plating 20,000 single cells/well into six-well ultra-low attachment dishes (Corning). Individual spheres ≥150 μm from each replicate well (n ≥ 9 wells) were counted under an inverted microscope at 50× magnification using the Image-Pro Plus program (Media Cybernetics). The percentage of cells capable of forming spheres, termed the ‘tumorsphere formation efficiency (TSFE)’, was calculated as follows: [(number of sphere formed/number of single cells plated) ×100].

### Cell proliferation assay

CT26 cells were seeded in 96-well plates. After 48 h of incubation, cell viability was assessed by cell counting kit-8 (Dojindo) according to the manufacturer’s instruction. The numbers of viable cells were measured at a wavelength of 450 nm using Versamax microplate reader.

### Real-time PCR

Total RNA was extracted using TRIzol reagent (Invitrogen). RNA purity was verified by measuring 260/280 absorbance ratio. The first strand of cDNA was synthesized with 1 μg of total RNA using SuperScript II (Invitrogen), and one-tenth of the cDNA was used for each PCR mixture containing Express SYBR-Green qPCR Supermix (BioPrince, Seoul, Korea). Real-time PCR was performed using a Rotor-Gene Q (Qiagen). The reaction was subjected to 40-cycle amplification at 95 °C for 20 sec, at 60 °C for 20 sec and at 72 °C for 25 sec. Relative mRNA expression of selected genes was normalized to HPRT and quantified using the DDCT method. The sequences of the PCR primers are listed in Table 1.

### Flow cytometry

FACS analysis and cell sorting were performed using FACS Calibur and FACS Aria machines (Becton Dickinson, Palo Alto, CA), respectively. FACS data were analyzed using Flowjo software (Tree Star, Ashland, OR). Antibodies to the following proteins were used: PE-conjugated CD44 (dilution 1/40). The FACS gates were established by staining with isotype antibody or secondary antibody.

### Protein isolation and western blot analysis

Protein expression levels were determined by western blot analysis as previously described^57^. Briefly, cells were lysed in a buffer containing 50 mM Tris, 5 mM EDTA, 150 mM NaCl, 1 mM DTT, 0.01% NP 40, 0.2 mM PMSF. The protein concentrations of the total cell lysates were measured by using bovine serum albumin (Sigma-Aldrich, St. Louis, MO) as standard. Samples containing equal amounts of protein were separated by sodium dodecyl sulfate polyacrylamide gel electrophoresis (SDS-PAGE) and then transferred onto polyvinylidene difluoride (PVDF) membranes (Bio-RAD Laboratories). The membranes were blocked with 5% skim milk in Tris buffered saline containing Tween-20 at RT, and the membranes were with primary antibodies overnight at 4 °C and then with HRP-conjugated secondary antibodies for 90 min at RT. Antibody-bound proteins were detected using an ECL.

### Immunofluorescent staining

Samples were fixed with 4% paraformaldehyde for fluorescent staining. Samples were permeabilized with 0.4 M glycine and 0.3% Triton X-100, and nonspecific binding was blocked with 2% normal swine serum (DAKO, Glostrup, Denmark). Staining was performed as described previously^58^, using the primary anti-Phalloidin (Cytoskeleton Inc.) antibody. Samples were examined by fluorescence microscopy (Zeiss LSM 510 Meta). The calculation of expression was based on green fluorescence area and density divided by cell number, as determined from the number of DAPI-stained nuclei, in three randomly selected fields for each sample from a total of three independent experiments.

### *In vitro* cell migration assay

Cell were plated at 1 × 10^5^ cells/well in 200 μL of culture medium in the upper chamber of Transwell permeable supports (Corning Inc, Corning, NY) with 8.0-μm pore, polycarbonate membrane, 6.5-mm diameter, and 24-well plate format) to track migration of CT26 cells. The cells on the upper surface of the membranes were completely removed by using a cotton swab. Migrated cells on the lower surface of the membranes were fixed with 4% paraformaldehyde for 10 min, stained with hematoxylin (Sigma-Aldrich), and later the number of cells was counted in three randomly selected fields of the wells under light microscope. To calculate the chemotactic index, the number of cells migrated in response to Id2 knockdown was divided by the number of spontaneously migrated cells (control).

### Metastasis/dissemination experiment

All animal experiments were approved and carried out in accordance with IACUC (Institutional Animal Care and Use Committee) guidelines (No.LCDI-2012-0069) of the Lee Gil Ya Cancer and Diabetes Institute. CT26 cells (2 × 10^5^) were subcutaneously injected into BALC mice, and tumor cell metastasis/dissemination was monitored. After cancer cell (CT26-Luc) injection, abdominal metastasis/dissemination was monitored using IVIS; the photon intensity, a measurement of viable tumor dissemination, was also determined by the system.

### Statistical analysis

All the statistical data were analyzed by GraphPad Prism 5.0 (GraphPad Software, San Diego, CA) and evaluated by two-tailed Student’s t-test. Value of P < 0.05 was considered to indicate statistical significance.

## Additional Information

**How to cite this article**: Dong, H.-J. *et al.* The Wnt/β-catenin signaling/Id2 cascade mediates the effects of hypoxia on the hierarchy of colorectal-cancer stem cells. *Sci. Rep.* **6**, 22966; doi: 10.1038/srep22966 (2016).

## Supplementary Material

Supplementary Information

## Figures and Tables

**Table 1:** Primer sequences for quantitative RT-PCR.

Gene	Genebank No.		Primer sequence
Mouse c-Myc	NM_010849	F	CGGACACACAACGTCTTGGAA
		R	AGGATGTAGGCGGTGGCTTTT
Mouse Oct4	NM_013633	F	GCATTCAAACTGAGGCACCA
		R	AGCTTCTTTCCCCATCCCA
Mouse Sox2	NM_011443	F	CAGCTGTCATTTGCTGTGGG
		R	AAATGGGAGGGGTGCAAAAG
Mouse Id1	NM_010495	F	CCCACTGGACCGATCCGCCA
		R	TGCTCTCGGTTCCCCAGGGG
Mouse Id2	NM_010496	F	TCTGGGGGATGCTGGGCACC
		R	GCTTGGGCATCTCCCGGAGC
Mouse Id3	NM_008321	F	CAGGGTCCCAAGCGAACGG
		R	TTGCCACTGACCCGGTCGTC
Mouse Id4	NM_031166	F	GGTGGCGGCTGTCTCAGCAA
		R	GCTCGTGCCTACCATCCCGC
Mouse β-catenin	NM_007614	F	TGGACCCTATGATGGAGCATG
		R	GGTCAGTATCAAACCAGGCCAG
Mouse MMP2	NM_013557	F	GCTGTATTCCCGACCGTTGA
		R	TGGTCCGCGTAAAGTATGGG
Mouse MMP9	NM_013558	F	AACATCTGGCACTCCACACC
		R	GCAGAAGTTCTTTGGCCTGC
Mouse Lef1	NM_010703	F	TGATTCCTGGTCCCCCTGGC
		R	CACTGTCCGTGTGGGGGTGC
Mouse Wnt1	NM_021279	F	GAACCCTTTTGCCATCCTGA
		R	CACCTTCAAGAGTTGACCTC
Mouse CyclinD1	NM_007631	F	AGGCAGCGCGCGTCAGCAGCC
		R	TCCATGGCGCGGCCGTCTGGG
Mouse Hprt	NM_013556	F	GCCTAAGATGAGCGCAAGTTG
		R	TACTAGGCAGATGGCCACAGG
Human Hif1a	NM_001530	F	TGCTCATCAGTTGCCACTTC
		R	TCCTCACACGCAAATAGCTG
Human TCF4	NM_030756	F	CGTAGACCCCAAAACAGGAA
		R	TCCTGTCCTTGATTGGGTACA
Human Id2	NM_002166	F	ATGAAAGCCTTCAGTCCCGT
		R	CGATCTGCAGGTCCAAGATG
Human c-Myc	NM_002467	F	AAAGGCCCCCAAGGTAGTTA
		R	GCACAAGAGTTCCGTAGCTG
Human Klf4	NM_004235	F	GAACTGACCAGGCACTACCG
		R	TTCTGGCAGTGTGGGTCATA
Human Nanog	NM_024865	F	ACATGCAACCTGAAGACGTGTG
		R	CATGGAAACCAGAACACGTGG
Human Oct4	NM_002701	F	ACATCAAAGCTCTGCAGAAAGAACT
		R	CTGAATACCTTCCCAAATAGAACCC
Human Wnt1	NM_005430	F	GCAGTGACAACATCGATTTTGG
		R	TCTTGGCGCATCTCAGAGAAC
Human CyclinD1	NM_053056	F	TGCATGTTCGTGGCCTCTAA
		R	TCGGTGTAGATGCACAGCTT
Human PPIA	NM_021130	F	TGCCATCGCCAAGGAGTAG
		R	TGCACAGACGGTCACTCAAA
