# Supplementary material for: The Wnt/β-catenin signaling/Id2 cascade mediates the effects of hypoxia on the hierarchy of colorectal-cancer stem cells
Source: Sci Rep. 2016 Mar 11;6:22966. doi: 10.1038/srep22966 (PMC4786801; doi:10.1038/srep22966)
Supplement: Supplementary Information [file srep22966-s1.pdf]

Supplementary information

Title: The Wnt/β-catenin signaling/Id2 cascade mediates the effects of hypoxia on the hierarchy of colorectal-cancer stem cells.

Authors: Hye-Jin Dong, Gyu-Beom Jang, Hwa-Yong Lee, Se-Ra Park, Ji-Young Kim, Jeong-Seok Nam, In-Sun Hong

Supplement Figure 1

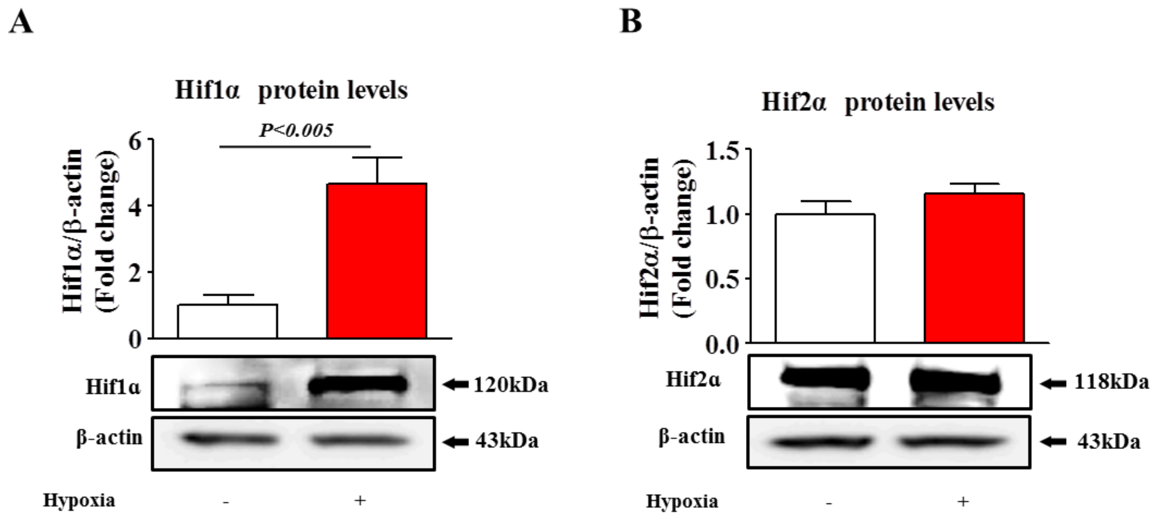

Supplementary figure 1. Effect of hypoxia on the expression of hypoxia-responsive genes. (A-B) The relative levels of expression of hypoxia-responsive, genes including HIF1α and HIF2α were assessed using western blotting. β-actin was used as the internal control. The results are presented as the mean values ± SD from three independent experiments.

Supplement Figure 2

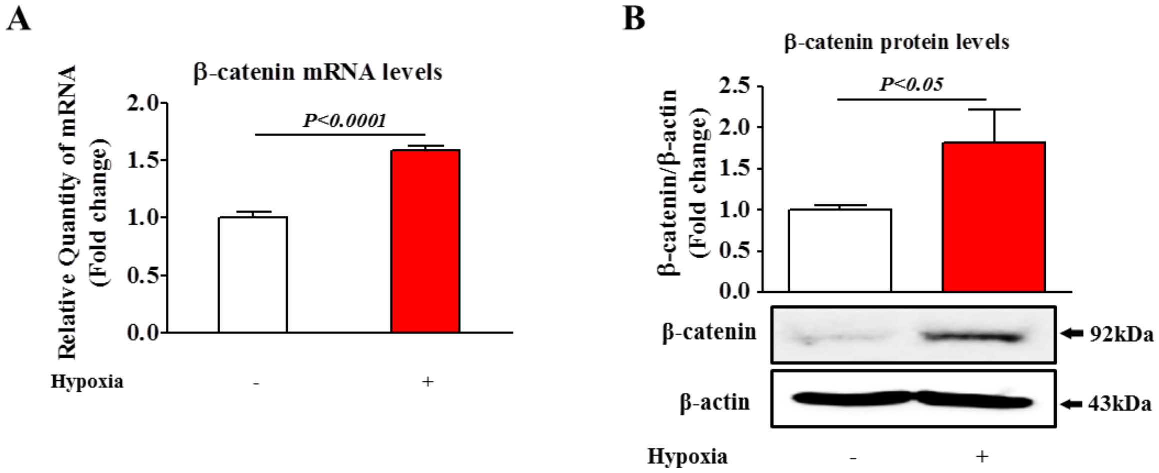

Supplementary figure 2. Effect of hypoxia on the expression of β-catenin. (A-B) Real-time PCR **(A)** and western blotting **(B)** demonstrated the hypoxia-induced changes in the expression of β-catenin. β-actin was used as the internal control. The results are the mean values ± SD from three independent experiments.

Supplement Figure 3

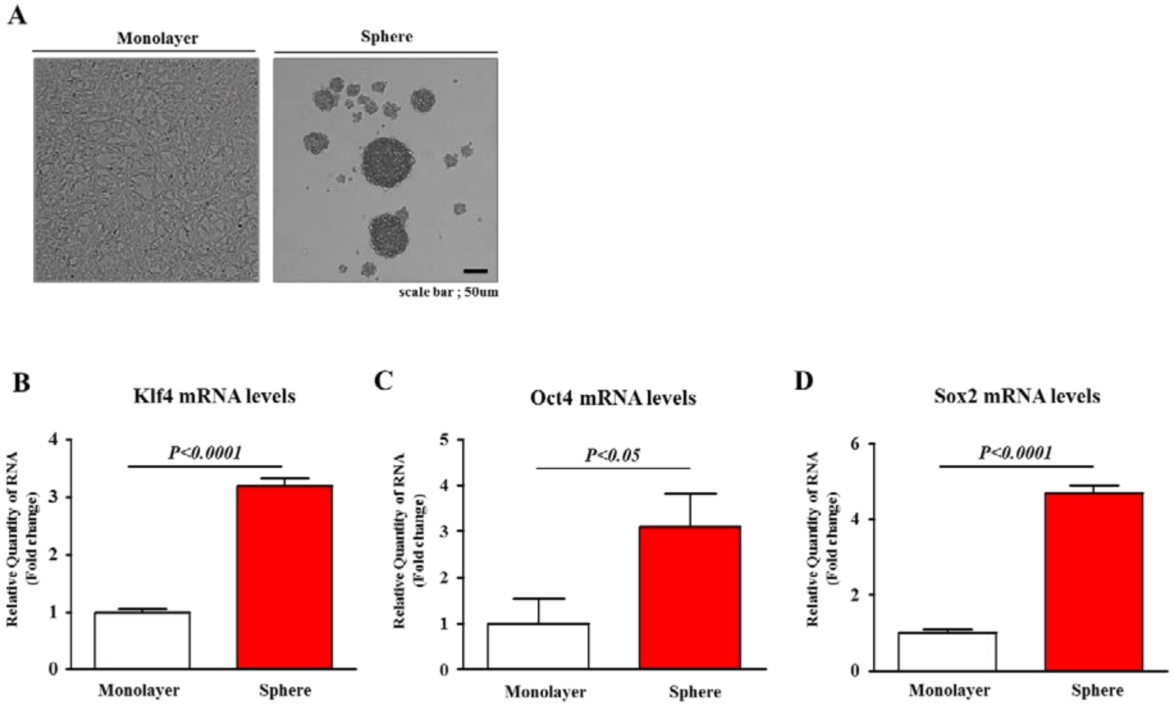

Supplementary figure 3. Culturing colorectal-cancer cell under sphere-forming conditions leads to the expression of stem-cell markers. Real-time PCR results demonstrating the changes in the expression of the stem-cell markers Klf4 **(B)**, Oct4 **(C)**, and Sox2 **(D)** after one week of sphere-formation culture relative to that of the cells in sub-confluent monolayers. The results are presented as the mean values ± SD from three independent experiments.

Supplement Figure 4

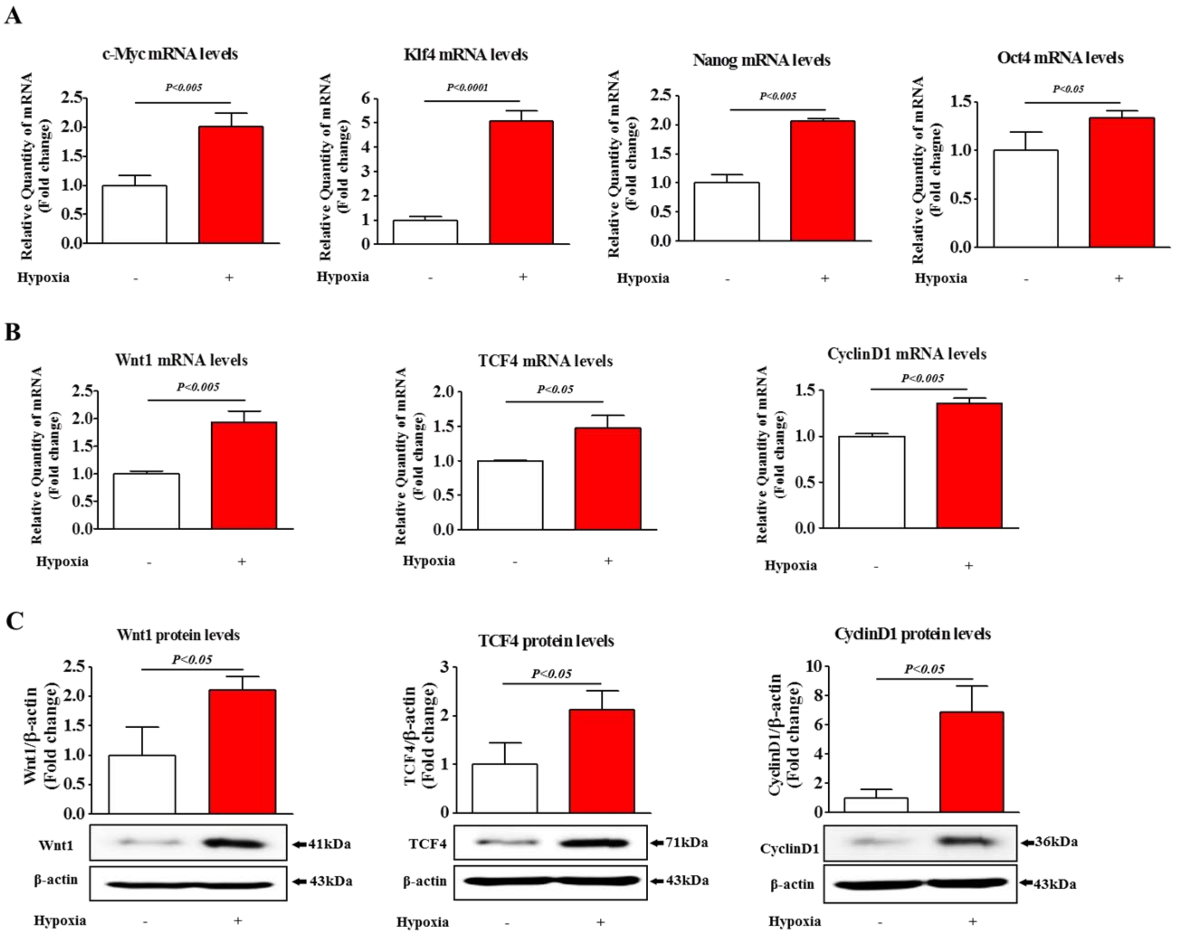

Supplementary figure 4. Effects of hypoxia on the expression of cancer stemness related factors and Wnt/β-catenin signaling components. (A) The expression levels of cancer stemness-related factors (c-Myc, Klf4, Nanog, and Oct4) under hypoxia were evaluated using real-time PCR. Real-time PCR **(B)** and western blotting **(C)** demonstrated the hypoxia-induced changes in the expression patterns of Wnt/β-catenin signaling components including Wnt1, TCF4, and Cyclin D1. β-actin was used as the internal control. The results are the mean values ± SD from three independent experiments.

Supplement Figure 5

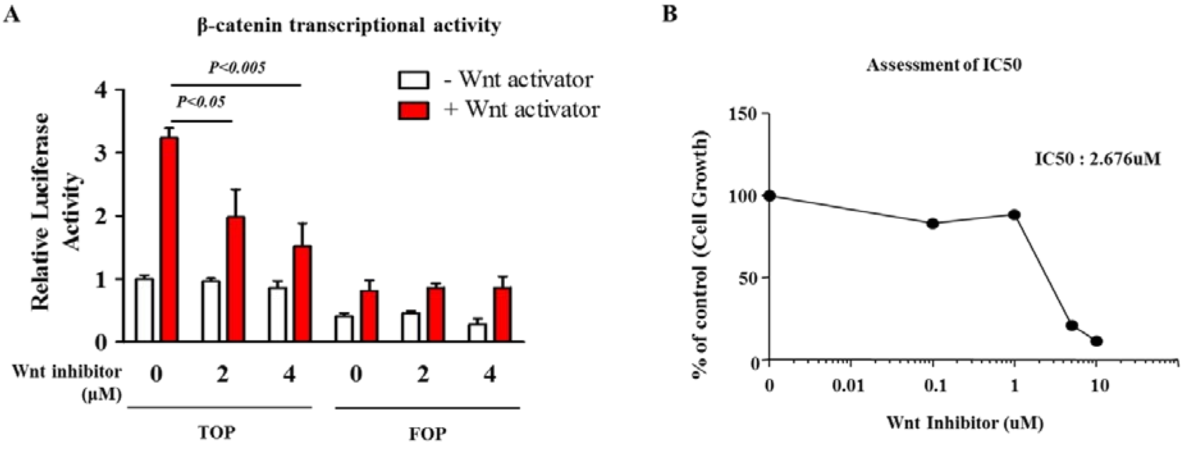

Supplementary figure 5. The efficacy and specificity of Wnt inhibitor to inhibit Wnt/β-catenin signaling. (A) β-catenin responsive TOPFlash luciferase assays revealed that Wnt inhibitor ICG-001 inhibits recombinant Wnt activator-induced Wnt/β-catenin signaling. Wnt inhibitor treatment strongly attenuated Wnt activator-induced TOPFlash activity. **(B)** The degree of decrease in the viability of the colorectal-cancer cells due to a 48-hour treatment with the Wnt/β-catenin signaling inhibitor ICG-001 was determined using the CCK-8 assay (which measured the level of mitochondrial-dehydrogenase activity). Cell viability (%) was calculated as the percentage of viable cells compared with that of cells treated with the vehicle control. The results are the mean values ± SD from three independent experiments.

Supplement Figure 6

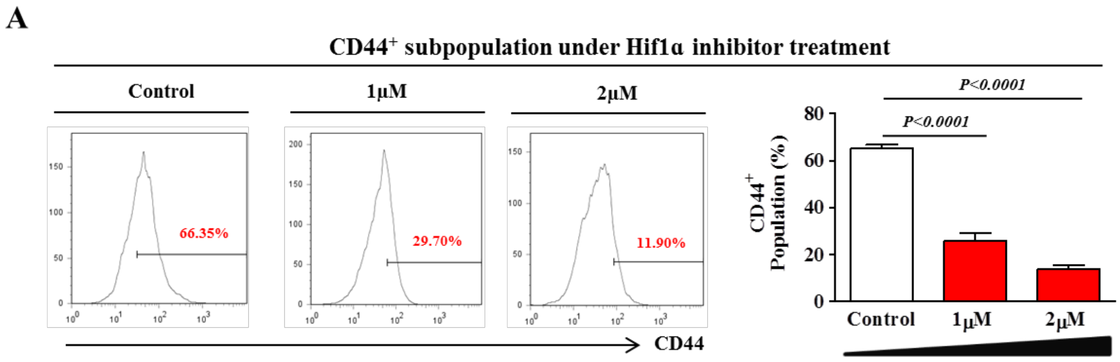

Supplementary figure 6. Inhibitory effect of HIF1α inhibitor on the percentage of the $CD44^+$ population (A) Treatment with a HIF1α inhibitor led to the decrease in the percentage of $CD44^+$ positive cells in the colorectal-cancer cells. The results are the mean values ± SD from three independent experiments.

Supplement Figure 7

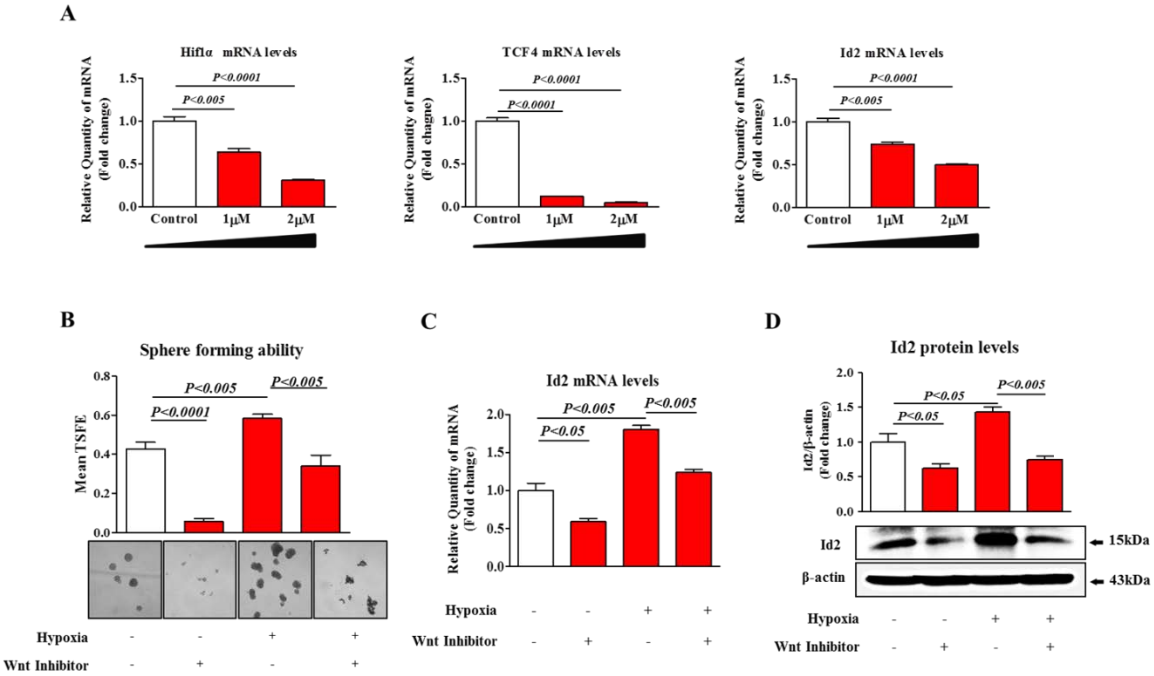

Supplementary figure 7. The potential role of hypoxia in Id2 expression and hypoxia-induced CSC-sphere formation in human colonic carcinoma epithelial cell line HCT116 (A) The results of real-time PCR demonstrated the mRNA levels of Hif1α, Wnt/β-catenin signaling component TCF4, and Id2 were significantly increased by HIF1α inhibitor treatment. **(B-D)** Pre-treating HCT116 cells with Wnt inhibitor ICG-001 significantly attenuated the hypoxia-induced sphere formation (**B**) and Id2 expression at both the mRNA **(C)** and protein **(D)** levels. β-actin was used as the internal control. The results are presented as the mean values ± SD from three independent experiments.

Supplement Figure 8

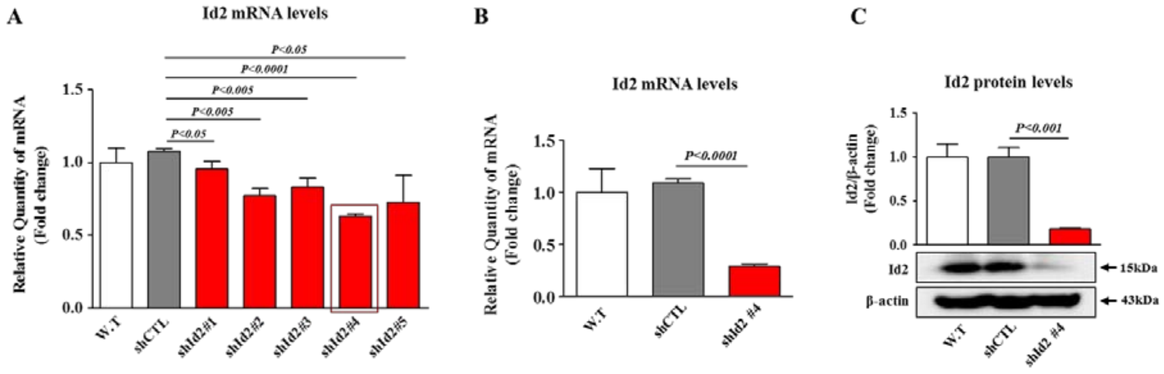

Supplementary figure 8. Knockdown efficacy of the shRNAs that targeted Id2 (A) Colorectal-cancer cells were stably transduced with shRNA #1, #2, #3, #4 or #5, which targeted Id2, or with a non-targeting control shRNA. Id2 shRNA construct #4, hereafter referred to as Id2 shRNA, was the most effective one. The successful knockdown of Id2 expression was verified based on the levels of RNA **(B)** and protein expression **(C)** in the colorectal-cancer cells. β-actin was used as the internal control. The results are presented as the mean values ± SD from three independent experiments.

Supplement Figure 9

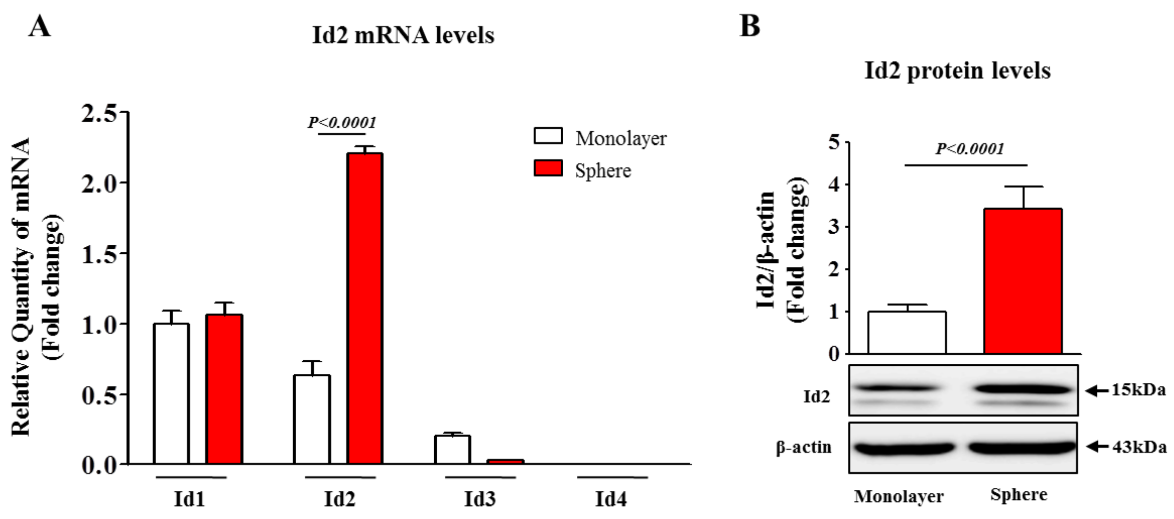

Supplemental figure 9. The level of Id2 expression was increased under the three-dimensional culture conditions. The relative level of Id2 expression in cells grown under the three-dimensional culture conditions was assessed using real-time PCR **(A)** and western blotting assays **(B)**. β-actin was used as the internal control. The results are presented as the mean values ± SD from three independent experiments.

Supplement Figure 10

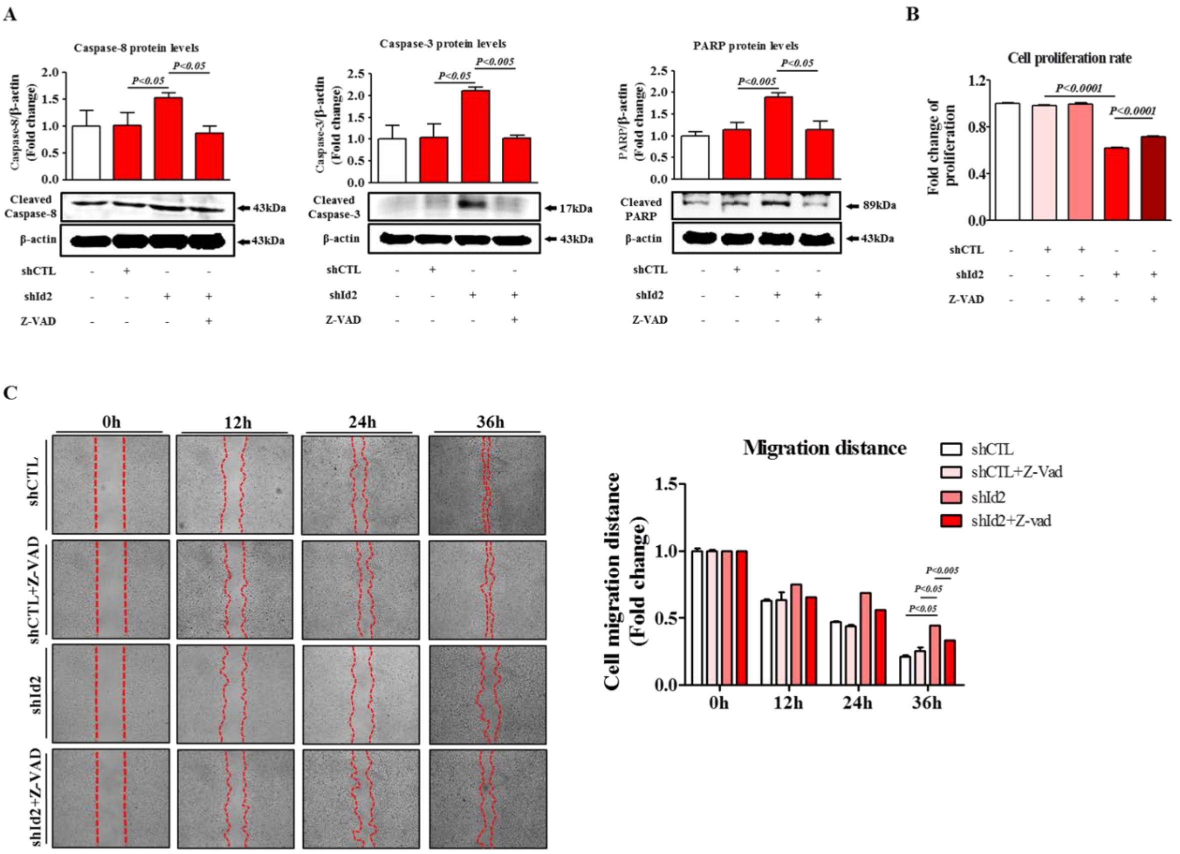

Supplemental figure 10. The effects of Id2 knockdown on the metastasis, cell proliferation, and migration were caused, or at least partially caused by Id2 shRNA-induced apoptosis. (A) CT26 cells were preincubated with a pancaspase inhibitor (Z-VAD-FMK) for 1 hr with or without ID2 knockdown; afterwards, cells were harvested for western blot analysis to detect cleaved caspase-8 and 3 and cleaved PARP. **(B-C)** Cell viability (%) was determined using the CCK-8 assay (which measured the level of mitochondrial-dehydrogenase activity). The effects of the Id2 knockdown on the migration of colorectal-cancer cells were evaluated using a scratch assay. Z-VAD-FMK pre-treatment attenuated Id2 knockdown-mediated effects on the proliferation (B) and migration (C). β-actin was used as the internal control. The results are presented as the mean values ± SD from three independent experiments.

Supplement Figure 11

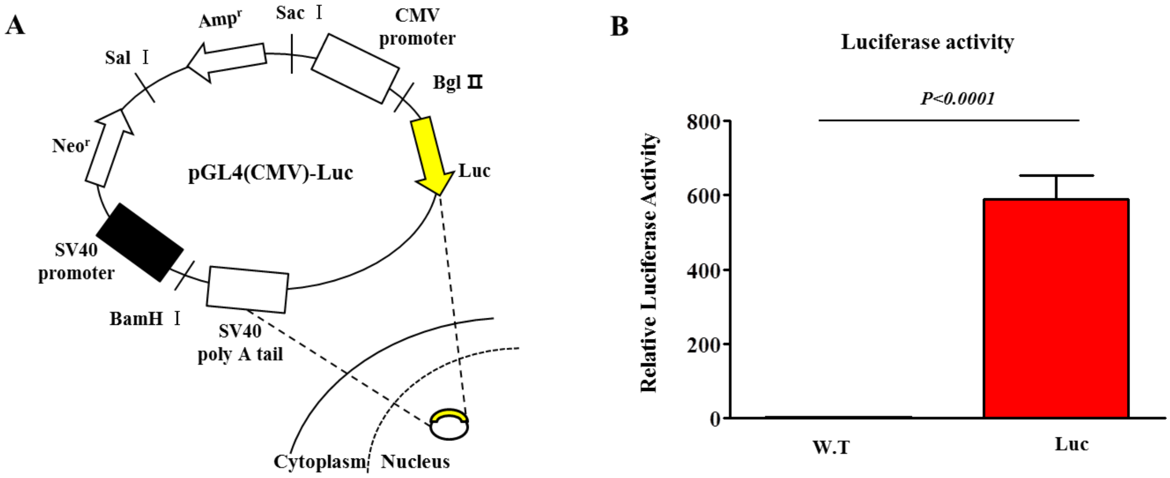

Supplemental figure 11. Establishment of a stable bioluminescent mouse colorectal-cancer cell line. (A-B) Colorectal-cancer cells were transfected with a pGL4 luciferase reporter vector. Luciferase assays revealed that a cell line stably expressing luciferase had been successfully generated. The results are presented as the mean values ± SD from three independent experiments.
